# Association between depression severity and trouble sleeping: A population-based study

**DOI:** 10.1097/MD.0000000000039611

**Published:** 2024-09-06

**Authors:** Yating Tu, Guangwei Qing, Meiying Chen, Haibo Chen

**Affiliations:** a Department of Psychiatry, Jiangxi Mental Hospital, Affiliated Mental Hospital of Nanchang University, Nanchang, Jiangxi, China.

**Keywords:** depression, NHANES, sleep disorder

## Abstract

This study investigates the association between insomnia and depression severity, exploring sleep disturbances in individuals with depression. The aim is to establish a new foundation for managing patients with co-occurring depression and insomnia, using 2015 to 2016 National Health and Nutrition Examination Survey (NHANES) data. We employed a cross-sectional design, using NHANES data from 2015 to 2018. The study included 11,261 participants after excluding incomplete data. Depression severity, assessed using Patient Health Questionnaire-9 (PHQ-9) scores, served as the exposure variable. We considered various demographic and lifestyle factors as covariates in the multivariate adjustment model. Statistical analyses adhered to CDC recommendations, with sample weights incorporated to account for NHANES’ complex sample design. Our study, encompassing 19,225 participants, revealed that higher PHQ-9 scores correlated with an increased likelihood of sleep disorders. In the fully adjusted model, a positive association emerged between PHQ-9 scores and trouble sleeping (OR = 3.95, 95% CI: 3.35–4.66, *P* < .0001). This relationship displayed an inverted U-shaped curve, with an inflection point at 28. Subgroup analysis and interaction tests indicated no reliance on factors such as gender, age, marital status, or BMI for the connection between depression severity and trouble sleeping (all *P* for interaction > .05). We identified a significant inverted U-shaped correlation between sleep disturbances and depression severity. This underscores the crucial importance of assessing sleep disorder risks in individuals with varying degrees of depression severity, facilitating personalized therapeutic interventions.

## 1. Introduction

Difficulties in falling asleep, which arise from the dysfunction of multiple regulatory systems, can display various adverse effects on an individual’s health and well-being. The most prevalent sleep-related affliction, insomnia, is characterized by trouble falling or staying asleep or poor sleep quality that leads to physical and psychological distress.^[1]^ Sleep disorders not only reduce daily productivity and quality of life, but they can also exacerbate the worst medical and psychiatric conditions.^[2]^ A recent study indicated that sleep disorders affect approximately 27.1% of American adults, and the incidence has alarmingly increased over the past few decades.^[3]^

Depression is a common mental disorder that is a leading cause of illness and disability and affects > 300 million individuals worldwide. The overall number of patients with depression rose by > 18% between 2005 and 2015.^[4]^ Thus, depression has far-reaching repercussions that include affecting academic and professional performances and disrupting positive family relationships.^[5]^ In Taiwan, depression accounted for 4.3% of the years lived with disability in 2019. The National Health Insurance spends approximately USD 1.342 billion on treating depression, and its incidence is increasing annually at a rate of 1.2%.^[6]^

Dysfunctional beliefs and attitudes about sleep are strongly associated with the occurrences of insomnia, stress, depression, anxiety, and suicidal ideation.^[7]^ The primary symptom of depression is impaired sleep, and depressed individuals often show alterations in sleep neurophysiology.^[8]^ A recent meta-analysis found that individuals with insomnia who do not have depression are twice as likely to have depression than those without sleep disturbances.^[9]^ Another study investigating insomnia has demonstrated its predictive value for depression.^[10]^ Although numerous studies have examined the potential association between insomnia and depression severity; however, the findings denoting this relationship need validation.

Hence, we investigated the correlation between depression severity, as measured by the Patient Health Questionnaire-9 (PHQ-9) score, and sleep disruption probability by utilizing the 2015 to 2016 National Health and Nutrition Examination Survey (NHANES) data to offer a novel basis for clinical management of patients with co-occurring insomnia and depression. This study was approved by the Ethics Committee of Jiangxi Mental Hospital.

## 2. Materials and methods

### 2.1. Ethics approval and consent to participate

The study was approved by the Jiang Xi Mental Hospital (Medical Research) Ethics Committee (No. 2024-006) in accordance with the Declaration of Helsinki. All methods were carried out in accordance with relevant guidelines and regulations.

### 2.2. Survey description

We employed a cross-sectional design and utilized NHANES data to examine the relationship between depression severity and the occurrence of sleep disturbances. NHANES is a comprehensive nationwide survey conducted by the National Center for Health Statistics (NCHS) that aims to evaluate the health and nutritional status of the U.S. population by using a complex multistage probability design.^[11]^ The comprehensive information regarding the NHANES study design and patient data can be accessed publicly at: www.cdc.gov/nchs/nhanes/.

### 2.3. Study population

**Figure 1.**
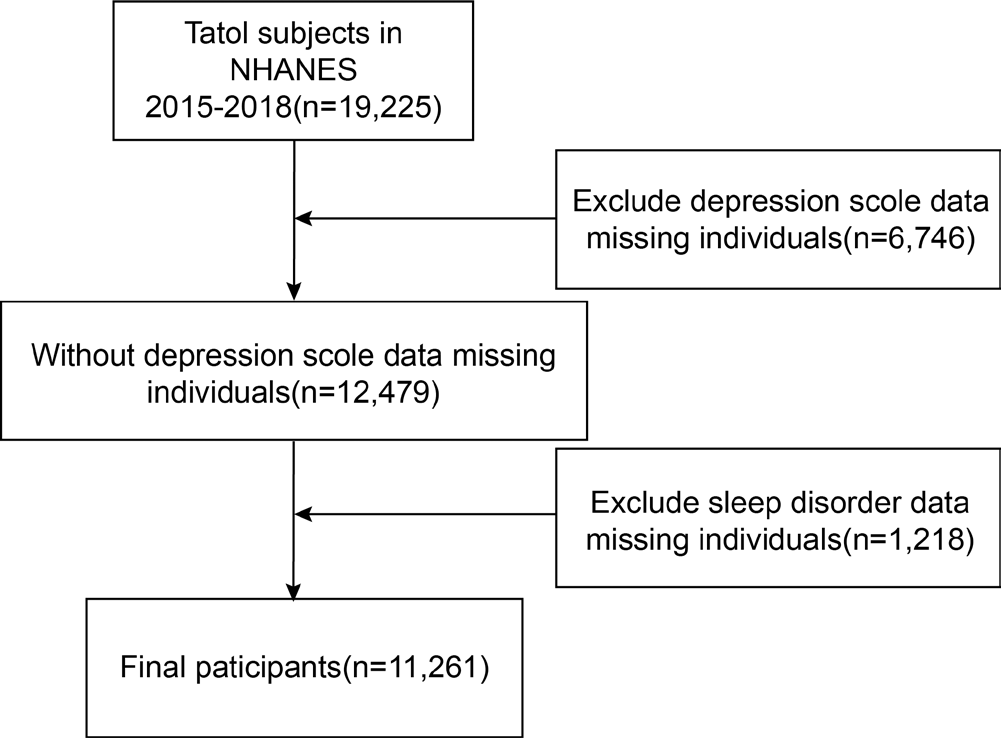
Flow chart of participants selection.

We used data from 2 consecutive NHANES surveys from 2015 to 2018. This nationally representative dataset provides comprehensive information regarding the incidence of depression as measured by the PHQ-9 scores and the prevalence of insomnia disorders. We initially enrolled 19,225 individuals; however, the removal of incomplete PHQ-9 scores (n = 2418) and incomplete insomnia data (n = 1218) data resulted in a sample of 11,261 participants (Fig. 1).

The NCHS Research Ethics Review Committee approved the NHANES study and all participants provided written informed consent.

We operationalized the depression severity exposure variable using (PHQ-9) scores. The PHQ-9 is a common screening tool for evaluating the severity of depressive symptoms and has demonstrated its effectiveness in many previous studies. The Diagnostic and Statistical Manual of Mental Disorders’ criteria are included in the questionnaire to assess the symptoms of depression. On a 4-point rating scale, all responses are rated from “0” (not at all) to “3” (nearly every day).^[12]^ Each participant’s PHQ-9 score, which ranged from 0 to 27, was determined by calculating the sum of all item scores. The PHQ-9 scoring system was used to determine depression severity; a score of < 10 indicated no depression, while a score of ≥ 10 indicated depression. A cutoff score of 10 showed good sensitivity and specificity for detecting major depression systems.^[13]^

“Have you/Has SP ever told a doctor or other health professional that you have/she/he has trouble sleeping?” was the question asked to the participants about their interactions with healthcare providers to assess the prevalence of trouble sleeping as an outcome variable. Thus, a positive response indicated difficulty falling asleep.^[14]^

### 2.4. Covariates

A multivariate adjustment model summarized the potential covariates that could affect the association between PHQ-9 scores and trouble sleeping, thereby minimizing potential confounding effects. Our study’s covariates were carefully selected based on previous research and clinical relevance. These covariates encompassed demographic factors such as age (years), gender, race (Mexican–American, Hispanic, non-Hispanic White, non-Hispanic Black, or others), BMI (kg/m^2^), waist circumference (cm), marital status (married, widowed, divorced, separated, never married, living with partner), education (<9th grade, high school/General Equivalent Diploma, some college or AA degree, college graduate or above), ratio of family income to poverty, diabetes (yes, no, borderline), smoking status, vigorous work activity, moderate work activity, vigorous recreation activities, moderate recreation activities, sleep hours, be sleepy (never, rarely, sometimes, often, almost always). All study variables were publicly accessible on the Centers for Disease Control and Prevention (CDC) website at www.cdc.gov/nchs/nhanes/.

### 2.5. Statistical analysis

All statistical analyses followed the CDC’s recommendations.^[15]^ All analyses were performed using Empower software (X&Y Solutions, Inc., Boston, MA, USA) and R version 3.4.3 (The R Foundation). We included appropriate sample weights in all analyses to adjust for the complex NHANES sample design. Continuous and categorical variables were reported as the mean with standard deviation or median with interquartile range and frequency or percentage, respectively. The differences between the with and without-trouble sleeping groups were assessed using a weighted Student *t* test or a chi-square test for continuous and categorical variables, respectively. We also used the penalized spline method for smooth curve fitting and weighted generalized additive model (GAM) regression for examining the potential nonlinear relationship between PHQ-9 score and trouble sleeping. Stratified multivariate regression analysis was conducted for variables such as age, gender, race, BMI, waist circumference, marital status, education level, the ratio of family income to poverty, diabetes, smoking status, vigorous work activity, moderate work activity, vigorous recreational activities, moderate recreational activities, sleep hours, and daytime sleepiness. The heterogeneous associations between the subgroups were also examined using an interaction term and log-likelihood ratio test model. All values of *P* < .05 were statistically significant.

## 3. Results

### 3.1. Participants’ baseline characteristics

Table 1 presents the participants’ demographic baseline characteristics. We included 11,261 individuals, with an average age of 48.91 ± 18.56 years, comprising 48.21% and 51.79% of males and females, respectively. Our results indicated that patients with depression (PHQ-9 scores ≥ 10) displayed an increased likelihood of insomnia compared to those without depression (PHQ-9 score < 10). Furthermore, individuals with insomnia were older, had a female predilection, non-Hispanic whites, obese, had attained some college education or an associate degree, divorced, diabetic, smoker, engaged in more moderate work activities and less vigorous recreational activities, experienced shorter sleep duration, and frequently reported daytime sleepiness as compared to those without sleep disorders.

**Table 1:** Population baseline table.

Trouble sleeping	No (N = 8199)	Yes (N = 3062)	Standardize diff	*P* value
Number of subjects	8199	3062		
Age (yr), mean ± SD	47.34 ± 18.82	53.12 ± 17.14	0.32 (0.28, 0.36)	<0.001
Sex, n (%)			0.15 (0.11, 0.19)	<0.001
Male	4121 (50.26)	1307 (42.68)		
Female	4078 (49.74)	1755 (57.32)		
Race, n (%)			0.29 (0.25, 0.33)	<0.001
Mexican American	1407 (17.16)	363 (11.85)		
Other Hispanic	937 (11.43)	332 (10.84)		
Non-Hispanic White	2455 (29.94)	1280 (41.80)		
Non-Hispanic Black	1819 (22.19)	688 (22.47)		
Other race	1581 (19.28)	399 (13.03)		
BMI (kg/m^2^), mean ± SD	28.93 ± 6.80	31.14 ± 8.19	0.29 (0.25, 0.34)	<0.001
Waist circumference (cm)	98.26 ± 16.26	104.19 ± 18.28	0.34 (0.30, 0.39)	<0.001
Marital status, n (%)			0.22 (0.17, 0.26)	<0.001
Married	4013 (51.77)	1380 (46.29)		
Widowed	545 (7.03)	278 (9.33)		
Divorced	727 (9.38)	446 (14.96)		
Separated	250 (3.23)	130 (4.36)		
Never married	1455 (18.77)	503 (16.88)		
Living with partner	761 (9.82)	244 (8.19)		
Education level (%)			0.13 (0.09, 0.18)	<0.001
<9th grade	855 (11.03)	254 (8.52)		
9 to 11th grade	893 (11.52)	348 (11.67)		
High school graduate/GED or equivalent	1770 (22.84)	667 (22.38)		
Some college or AA degree	2288 (29.52)	1033 (34.65)		
College graduate or above	1945 (25.09)	679 (22.78)		
Ratio of family income to poverty	2.46 ± 1.61	2.46 ± 1.60	0.00 (-0.04, 0.05)	0.903
Diabetes, n (%)			0.26 (0.22, 0.30)	<0.001
Yes	987 (12.04)	657 (21.46)		
No	7016 (85.57)	2310 (75.44)		
Borderline	196 (2.39)	95 (3.10)		
Smoking status, n (%)			0.29 (0.25, 0.34)	<0.001
Yes	2991 (36.48)	1558 (50.88)		
No	5208 (63.52)	1504 (49.12)		
Vigorous work activity, n (%)			0.03 (-0.01, 0.08)	0.342
Yes	1859 (22.67)	660 (21.55)		
No	6340 (77.33)	2402 (78.45)		
Moderate work activity, n (%)			0.08 (0.04, 0.13)	<0.001
Yes	3177 (38.75)	1310 (42.78)		
No	5022 (61.25)	1752 (57.22)		
Vigorous recreational activities, n (%)			0.19 (0.15, 0.23)	<0.001
Yes	2235 (27.26)	595 (19.43)		
No	5964 (72.74)	2467 (80.57)		
Moderate recreational activities, n (%)			0.05 (0.00, 0.09)	0.071
Yes	3354 (40.91)	1184 (38.67)		
No	4842 (59.9)	1878 (61.33)		
Sleep hours	7.71 ± 1.57	7.58 ± 1.77	0.08 (0.04, 0.12)	0.004
Be sleepy, n (%)			0.54 (0.50, 0.58)	<0.001
Never	1668 (20.34)	302 (9.86)		
Rarely	2134 (26.03)	487 (15.90)		
Sometimes	2733 (33.33)	1004 (32.80)		
Often	1178 (14.37)	802 (26.19)		
Almost always	486 (5.93)	467 (15.25)		
Depression score, n (%)			0.51 (0.46, 0.55)	<0.001
<10	7803 (95.17)	2408 (78.64)		
≥10	396 (4.83)	654 (21.36)		

Abbreviations: BMI = body mass index, PHQ-9 = Patient Health Questionnaire-9.

### 3.2. Association between depression severity and sleep disorders

A significant positive correlation was observed between PHQ-9 score and sleep disturbances in depressed individuals (PHQ-9 score ≥ 10). Model 2 adjusted for various confounding variables such as age, gender, race, BMI, waist circumference, marital status, education level, the ratio of family income to poverty, diabetes, smoking status, vigorous work activity, moderate work activity, vigorous recreational activities, moderate recreational activities, sleep duration, and daytime sleepiness. A significant association was noticed between PHQ-9 score and trouble sleeping (OR = 3.95, 95% CI: 3.35–4.66, *P* < .0001) even after controlling potential confounders. An increase of 1 unit in the PHQ-9 score is linked to a 2.95-fold increase in the likelihood of having difficulty sleeping among depressed patients (PHQ score ≥ 10) (Table 2).

**Table 2:** Association between depression score and sleep disorders in multiple regression model.

Variable	Crude model OR (95% CI)	*P* value	Model I OR (95% CI)	*P* value	Model II OR (95% CI)	*P* value
Depression score < 10	1.0		1.0		1.0	
Depression score ≥ 10	5.35 (4.69, 6.11)	<.0001	5.30 (4.62, 6.07)	<.0001	3.95 (3.35, 4.66)	<.0001

Crude model: No other covariates were adjusted. Model I: adjusted for age, gender and race. Model II: adjusted for age, gender, race, BMI, waist circumference, marital status, education level, ratio of family income to poverty, diabetes, smoking status, vigorous work activity, moderate work activity, vigorous recreational activities, moderate recreational activities, sleep hours and be sleepy.

### 3.3. Nonlinear inverted U-shaped association of PHQ-9 score and trouble sleeping in U.S. adults

**Figure 2.**
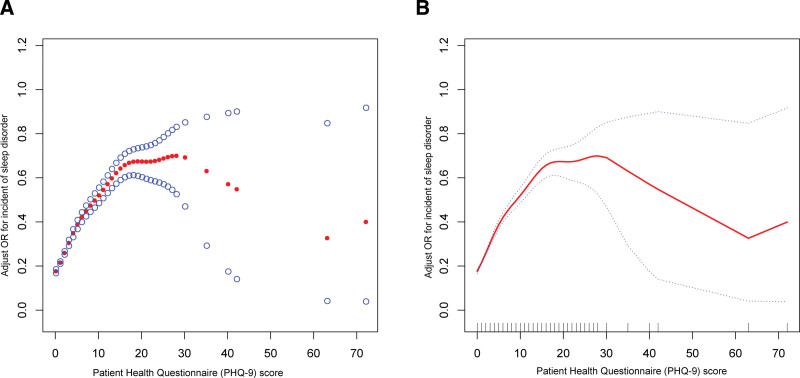
Nonlinear inverted U-shaped association of PHQ-9 score and trouble sleeping.

Weighted GAMs and smooth curve fittings helped in investigating the potential nonlinear association between PHQ-9 scores and sleep disturbances (Fig. 2). Since a nonlinear curve fitting analysis indicated an inverted U-shaped relationship between PHQ-9 score and trouble sleeping, a breakpoint analysis investigated the nonlinear relationship between PHQ-9 score and trouble sleeping and revealed a breakpoint score of 28. We also observed a positive association (OR = 1.14, 95% CI: 1.13–1.16) between PHQ-9 scores and trouble sleeping when the score was < 28, indicating that higher PHQ-9 scores were linked to an increased likelihood of insomnia. Conversely, a negative association (OR = 0.92, 95% CI: 0.87–0.98) was observed when this score > 28, indicating that higher PHQ-9 scores were linked to reduced insomnia cases. Thus, these findings suggest that the complex relationship between PHQ-9 scores and insomnia highlights the need for clarifying the underlying mechanisms. Additionally, a tailored approach based on an individual’s PHQ-9 score might be beneficial for managing depression and sleep disturbances. The results of the inflection points are shown in Table 3.

**Table 3:** Threshold effect analysis of PHQ-9 score on trouble sleeping in adults using 2-piecewise linear.

Trouble sleeping	Adjusted OR (95% CI)
Fitting by standard linear model	1.14 (1.12–1.15) < 0.0001
Fitting by 2-piecewise linear model	
Inflection point	28
PHQ-9 score < 27	1.14 (1.13–1.16) < 0.0001
PHQ-9 score > 27	0.92 (0.87–0.98) 0.0149
Log likelihood ratio	<0.001

Age, gender, race, BMI, waist circumference, marital status, education level, ratio of family income to poverty, diabetes, smoking status, vigorous work activity, moderate work activity, vigorous recreational activities, moderate recreational activities, sleep hours and be sleepy were adjusted.

### 3.4. Subgroup analysis

A subgroup analysis was undertaken to evaluate the robustness and validity of the correlation between PHQ-9 scores and insomnia. Furthermore, we investigated potential effect modifications by examining interactions with gender, age, marital status, and BMI and revealed that none of these interactions were statistically significant (all *P*-values for interaction > .05). Thus, the correlation between PHQ-9 score and trouble sleeping was not dependent on gender, age, marital status, or BMI. Our results demonstrated the robustness of the relationship between PHQ-9 scores and insomnia across diverse demographic and clinical variables like gender, age, marital status, and BMI, thereby indicating its potential generalizability to broader demographic settings (Table 4).

**Table 4:** Association between depression score and sleep disorder in subgroups.

Subgroup		OR (95%CI)	*P* for interaction
Sex			.5995
Male	N = 4306	1.13 (1.11, 1.15)	
Female	N = 4565	1.14 (1.12, 1.16)	
Age(years)			.8515
<30	N = 1448	1.12 (1.09, 1.16)	
30 to 65	N = 5345	1.14 (1.12, 1.16)	
>65	N = 2078	1.12 (1.10, 1.15)	
Marital status			.0532
Married	N = 4538	1.15 (1.13, 1.18)	
Others	N = 4333	1.13 (1.11, 1.14)	
BMI (kg/m^2^)			.8681
<25	N = 2395	1.14 (1.12, 1.17)	
25 to 30	N = 2828	1.13 (1.10, 1.15)	
>30	N = 3648	1.14 (1.12, 1.16)	

Stratified analyses assessing the association between depression score and sleep disorder. Results are presented as adjusted ORs (95% CI) of depression score, which were adjusted for age, gender, race, BMI, waist circumference, marital status, education level, ratio of family income to poverty, diabetes, smoking status, vigorous work activity, moderate work activity, vigorous recreational activities, moderate recreational activities, sleep hours, be sleepy.

## 4. Discussion

This study was to evaluate the association between PHQ-9 score and trouble sleeping among U.S. adults. In our cross-sectional study with 11,261 participants, we observed that participants with higher PHQ-9 scores showed an increased likelihood of trouble sleeping. Our analysis revealed a nonlinear association between PHQ-9 scores and sleep disorders, characterized by an inverted U-shaped relationship. This suggested that the complex relationship between PHQ-9 score and sleep disorders requires careful consideration in clinical management. Notably, it is notable to mention that the observed positive correlation was statistically significant across various subgroups (all *P*-values < .05).

To the best of our knowledge, this is the first cross-sectional study that examined the correlation between depression severity and sleep disturbances. Although many previous studies have explored this association, Cameron et al in their cross-sectional study of 4700 Mexican–American adults, revealed that higher depression severity was linked with a greater probability of insufficient sleep and oversleeping. Furthermore, for each incremental increase in PHQ-9 score, the likelihood of insufficient sleep and oversleeping also increased.^[16]^ In a prospective study by Robert et al involving 4175 adolescents aged 11 to 17 years, it was discovered that decreased sleep quantity was associated with an increased risk of major depression, which in turn increased the risk of sleep deprivation. Additionally, sleep deprivation raised the risk of subsequent major depression by more than threefold.^[17]^ Nyer et al evaluated a cohort of 287 college students who exhibited depressive symptoms (as indicated by a Beck Depression Inventory score ≥ 13). Nevertheless, no statistically significant relationship was observed between depression severity and sleep disorders.^[9]^ In a cross-sectional study conducted by Taylor et al on 373 college students with a mean age of 21 years, people with insomnia had increased depression severity in comparison to people without insomnia (PWOI) symptoms.^[18]^ Correspondingly, we also observed a significant correlation between depression severity and the likelihood of sleep disorders, which followed an inverted U-shaped relationship and exhibited a positive trend when the PHQ-9 score was < 28.

Since depression and sleep disturbances are interrelated, their underlying physiological mechanisms have been extensively studied. Depressed patients usually exhibit findings like abnormal sleep EEG recordings, reduced sleep efficiency, decreased NREM sleep, and increased REM sleep duration. REM sleep disturbances are a hallmark of depression and have been observed in animal models of depression, suggesting that REM sleep abnormalities might serve as a potential biomarker for depression.^[19]^ Regarding biorhythmic variables, the internal coincidence model posits that depressed patients suffer from an incorrect sleep schedule due to a misalignment of the phase angle between their biological clock and sleep-wake cycle. Thus, prolonged asynchrony between these 2 systems can contribute to the development of depressive disorders.^[20–22]^ A study revealed that individuals with severe depressive and anxiety symptoms display limited physical activity, longer sleep duration, and lower RA between day and night activity levels.^[23]^ Exercise, behavioral activation, and chronotherapy are examples of supplementary therapies that may be used with traditional therapy since both physical activity and psychological recovery are modifiable factors.^[24,25]^ A recent study reported a correlation between depression severity and circadian rhythm disruptions. Specifically, the degree of insomnia in severely depressed patients was correlated with the asynchronous relationship between dim light melatonin onset and the nadir of core body temperature.^[26,27]^ Hickie et al postulated that a constellation of symptoms, including insomnia and depression, could be caused by disruptions in the intricate interactions among various physiological cycles, such as core body temperature, melatonin and cortisol plasma concentrations, sleep-wake timing, and other related factors.^[28]^ The possibility of an inverse U-shaped association has been discussed cautiously, despite the fact several studies have demonstrated a clear relationship between depression severity and sleep disturbance.

Thus, the following inferences can be drawn: patients may have insomnia, and nocturnal as well as early morning awakenings due to enhanced negative emotional states.^[26,29]^ An escalation in depression severity leads to an increased likelihood of sleep disorders. However, sleep disturbances might decrease beyond a certain threshold of symptoms because of a state of exhaustion and reduced reactivity to external stimuli, which makes falling asleep easier. Circadian rhythm disruption might cause sleep disturbances in patients with depression.^[30,31]^ Depressed patients often experience irregular sleep-wake cycles. Nonetheless, the worsening of symptoms increases the degree of circadian rhythm disruption, leading to more severe sleep disturbances. Moreover, the circadian rhythm may be completely disrupted, resulting in reduced sleep disturbances once symptoms intensify to a certain degree. Endocrine system dysregulation in depressed patients may lead to abnormal secretions of melatonin and cortisol and contribute to the onset of sleep disturbances.^[32–34]^ Sleep disturbances may occur as a result of irregularities in melatonin and cortisol secretions, which might worsen along with depressive symptoms.^[35–37]^ However, once symptoms reach a certain level, the endocrine system might reach a new equilibrium and reduce the occurrence of sleep disturbances.

Our study exhibits several strengths. Firstly, our research data was based on the NHANES database, which is a nationally representative population-based sample survey, having a large sample size. Additionally, subgroup analyses stratified by gender, age, marital status, and body mass index (BMI) demonstrated that this association was accurate for diverse demographic situations. However, our study had several limitations. Our study’s cross-sectional design could not help in establishing a definitive causal relationship. Furthermore, our inaccurate results might be due to our inability to account for all potential covariates that might have influenced depression severity and sleep disorders. Moreover, this association might have been affected by antidepressant usage (e.g., agomelatine) by depressed patients, which modulates melatonin changes via MT1/MT2 melatonin receptors and 5-HT2C serotonin receptors to affect circadian rhythms. However, these confounding factors were not considered in the NHANES data.^[38–41]^ Therefore, our study findings warrant careful interpretation in future studies.

## 5. Conclusion

We found a substantial inverted U-shaped connection between the occurrence of sleep disturbances and depression severity in our cross-sectional study comprising 11,261 adult participants. Thus, these results highlight the critical need to assess sleep disorder risks in patients with varying degrees of depression severity, thereby facilitating customized therapeutic interventions. Nonetheless, more future prospective studies and clinical settings are needed to substantiate these observations.

## Acknowledgments

We thank all the participants in the study.

## Author contributions

**Conceptualization:** Yating Tu.

**Data curation:** Guangwei Qing.

**Investigation:** Meiying Chen.

**Methodology:** Yating Tu.

**Software:** Yating Tu.

**Writing – original draft:** Yating Tu, Guangwei Qing.

**Writing – review & editing:** Meiying Chen, Haibo Chen.
